# The effect of ADAMTS13 on graft‐versus‐host disease

**DOI:** 10.1111/jcmm.18457

**Published:** 2024-07-04

**Authors:** Dan Li, Min Soon Cho, Ricardo Gonzalez‐Delgado, Xiaowen Liang, Jing‐Fei Dong, Miguel A. Cruz, Qing Ma, Vahid Afshar‐Kharghan

**Affiliations:** ^1^ Department of Hematopoietic Biology & Malignancy The University of Texas MD Anderson Cancer Center Houston Texas USA; ^2^ Section of Benign Hematology The University of Texas MD Anderson Cancer Center Houston Texas USA; ^3^ Department of Integrative Biology and Pharmacology McGovern Medical School, The University of Texas Health Science Center Houston Texas USA; ^4^ Bloodworks Research Institute and Hematology Division, Department of Medicine University of Washington School of Medicine Seattle Washington USA; ^5^ Center for Translational Research on Inflammatory Diseases (CTRID), Michael E. DeBakey VA Medical Center Houston Texas USA; ^6^ Baylor College of Medicine Houston Texas USA; ^7^ Department of Medicine Baylor College of Medicine Houston Texas USA

**Keywords:** ADAMTS13, graft‐versus‐host disease, Von Willebrand factor

## Abstract

Allogeneic haematopoietic stem cell transplantation (allo‐HSCT) can potentially cure malignant blood disorders and benign conditions such as haemoglobinopathies and immunologic diseases. However, allo‐HSCT is associated with significant complications. The most common and debilitating among them is graft‐versus‐host disease (GVHD). In GVHD, donor‐derived T cells mount an alloimmune response against the recipient. The alloimmune response involves several steps, including recognition of recipient antigens, activation and proliferation of T cells in secondary lymphoid organs, and homing into GVHD‐targeted organs. Adhesion molecules on T cells and endothelial cells mediate homing of T cells into lymphoid and non‐lymphoid tissues. In this study, we showed that Von Willebrand factor (VWF), an adhesion molecule secreted by activated endothelial cells, plays an important role in mouse models of GVHD. We investigated the effect of the VWF‐cleaving protease ADAMTS13 on GVHD. We found that ADAMTS13 reduced the severity of GVHD after bone marrow transplantation from C57BL6 donor to BALB/C recipient mice. A recombinant VWF‐A2 domain peptide also reduced GVHD in mice. We showed that ADAMTS13 and recombinant VWF‐A2 reduced the binding of T cells to endothelial cells and VWF in vitro, and reduced the number of T cells in lymph nodes, Peyer's patches and GVHD‐targeted organs in vivo. We identified LFA‐1 (αLβ2) as the binding site of VWF on T cells. Our results showed that blocking T‐cell homing by ADAMTS13 or VWF‐A2 peptide reduced the severity of the GVHD after allo‐HSCT, a potentially novel method for treating and preventing GVHD.

## 1 INTRODUCTION

Graft‐versus‐host disease (GVHD) is the most common cause of morbidity and mortality after allogeneic haematopoietic stem cell transplantation (allo‐HSCT)^1^, ^2^ and affects, to various degrees, about 70% of recipients. GVHD is a multistep process involving different cells.^3^ Infused donor T cells migrate and home to secondary lymphoid structures (spleen, lymph node and Peyer's patch) and are exposed to host antigen‐presenting cells (priming). Donor T cells activated by host alloantigens move to target organs such as the intestines, liver and skin and mount an immune response causing tissue injury. Migration and homing of donor T cells to lymphoid and non‐lymphoid organs require the interaction between T cells and endothelial cells mediated by selectins and integrins on T cells and their ligands on endothelial cells.^4^ The binding of adhesion molecules and activation of T cells by chemokines (most importantly CCL21, also known as secondary lymphoid tissue chemokine^5^) result in tethering, rolling, firm adhesion and extravasation of T cells.^6^ L‐selectin on T cells binds to sialyl lewis‐X carrying addressins (peripheral node addressin or mucosal addressin) on endothelial cells, and integrins (αLβ2 and α4β7) on T cells bind to their ligands on endothelial cells. Like lymphocytes, neutrophils and platelets also require integrins for firm adhesion to their ligands on the endothelial cells. Platelets' GPIb‐IX‐V complex and integrin αIIbβ3 bind to Von Willebrand factor (VWF) anchored on activated endothelial cells for rolling and firm adhesion, respectively. Endothelial cell‐bound VWF also recruits leukocytes by either directly binding to leukocytes or indirectly to platelet–leukocyte aggregates.^7^ VWF is a multimeric plasma protein that is secreted by activated endothelial cells and platelets. A mature VWF monomer is a large protein with multiple domains arranged as D′‐D3‐A1‐A2‐A3‐D4‐C1‐C6‐Cystine knot.^8^ The A2 domain of VWF (VWF‐A2) contains a proteolytic site for the metalloprotease ADAMTS13 (a disintegrin and metalloprotease with thrombospondin motif), a member of the ADAMTS family.^9^ Newly secreted unusually large VWF multimers (ULVWF) are cleaved on the surface of endothelial cells by ADAMTS13.^9^, ^10^, ^11^, ^12^, ^13^

VWF is an acute phase reactant whose plasma level increases upon release from stimulated endothelial cells in inflammation. The VWF increase is associated with a relative decline in ADAMTS13 function in plasma.^14^, ^15^ An increase in VWF, in turn, exacerbates the inflammation by recruiting neutrophils and monocytes to the site of inflamed vessels in infection, cerebral ischemia, myocardial ischemia and atherosclerosis models.^16^, ^17^, ^18^ On the other hand, cleavage of VWF by ADAMTS13 reduced vascular inflammation by reducing the migration of neutrophils, monocytes and T cells to tissue.^19^, ^20^, ^21^, ^22^, ^23^, ^24^

In this study, we showed that ADAMTS13 reduced the severity of acute GVHD in a mouse model of bone marrow transplantation. Recombinant VWF‐A2 had the same anti‐GVHD effect. The decrease in donor T cells in secondary lymphoid organs and parenchyma of GVHD‐targeted organs indicates the possibility of ADAMTS13‐ and VWF‐A2 peptide‐induced reduction in homing of T cells. We showed that Jurkat cells bind to histamine‐stimulated endothelial cells and VWF in vitro, and this binding was reduced by ADAMTS13 and recombinant VWF‐A2 peptide. Due to the importance of integrin αLβ2 in T‐cell adhesion, we examined protein–protein interaction between VWF and αL by surface plasmon resonance and detected αL binding to immobilized VWF. Furthermore, we showed that VWF‐A2 binds to immobilized αL. Our results demonstrate the role of VWF in T‐cell homing and the pathogenesis of GVHD, and the potential therapeutic benefit of interrupting T‐cell migration by administrating ADAMTS13 or recombinant VWF‐A2 peptide.

## 2 METHODS

### 2.1 Reagents

The monoclonal antibodies specific to mouse H‐2D^b^ (KH95), CD45 (30‐F11), CD3 (145‐2C11), CD4 (RM4‐5), CD8 (53–6.7), CD25 (7D4), Foxp3 (3G3), IL‐4 (11B11), IFN‐γ (XMG1.2), IL‐17 (TC11‐18H10.1), IL‐2 (JES6‐5H4), TNF‐α (MP6‐XT22), B220 (RA3‐6B2), CD11c (N418), CD11b (M1/70), Ly6C (HK1.4), Ly6G (1A8), NK‐1.1 (PK136) and F4/80 (BM8) were from BD Biosciences (San Diego, CA) and Biolegend (San Diego, CA). For intracellular cytokine production analysis, cells were diluted into 1 × 10^6^ cells/ml in RPMI‐1640 medium supplemented with 10% FBS and 1% penicillin–streptomycin (1,000 units/mL penicillin and 100 mg/mL streptomycin) (Thermo Fisher Scientific, Waltham, MA). Cells were stimulated with the Leukocyte Activation Cocktail with GolgiPlug (BD Biosciences, San Diego, CA) and then fixed and permeabilized with Fix & Perm A/B buffer from Life Technologies (Carlsbad, CA). Jurkat immortalized human T cells were purchased from ATCC (Manassas, VA). Recombinant ADAMTS13 was purchased from Bio‐techne/Tocris (Minneapolis MN), and recombinant VWF‐A2 protein was produced, as described previously.^25^ C57BL/6 and BALB/c mice were purchased from Jackson Lab.

### 2.2 The complete blood count and the flow cytometric immune profiling of BALB/c mice before and after administration of VWF‐A2 and ADAMTS13

Healthy female 6–8‐week‐old BALB/c (H‐2Dd) mice were treated with ADAMTS13 or VWF‐A2 to test their effect on immune cells. A group of BALB/c mice received recombinant VWF‐A2, another group received recombinant ADAMTS13 (rADAMTS13), and the control group received buffer (PBS). VWF‐A2 was administrated intraperitoneally (i.p.) at the dose of 3 mg/kg on Day 0, Day 2, Day 4 and Day 6. ADAMTS13 was administrated via the tail vein (i.v.) at a dose of 300 μg/kg on Day 0 and Day 5. The control group for each experiment received PBS at the same time and through the same route as the experimental reagent. Twenty‐five μl of blood was collected from each mouse on Day 0 before treatment (basal level) and on Day 4 and Day 7 after treatment. The complete blood count was performed on Scil Vet abc Plus™ from Scil Animal Care Company GmbH (Viernheim, Germany). Fifty μl of blood was also collected from each mouse on Day 0 before treatment (basal level) and on Day 4 and Day 7 after treatment and used for the flow cytometric immune profiling. Blood was first lysed for red blood cells and then stained with CD45, CD3, CD4, CD8, CD25, Foxp3, B220, CD11c, CD11b, Ly6C, Ly6G, NK‐1.1 and F4/80 antibodies. T cells (CD3^+^CD4^+^, CD3^+^CD8^+^, CD3^+^ CD4^+^CD25^+^Foxp3^+^), B cells (CD3^−^B220^+^), monocytes (CD3^−^CD11b^+^ Ly6C^hi^), NK cells (CD3^−^NK‐1.1^+^), macrophages (CD3^−^F4/80^+^), neutrophils (CD11b^+^ Ly6G^+^) and DC (CD3^+^CD11c^+^) were detected and analyzed. Mice were sacrificed on Day 7 after treatment. Cells were collected from the spleen (SP) and peripheral lymph nodes (LN) and stained with listed antibodies. Each immune cell population was collected and analysed by using a BD LSRFortessa X‐20 flow cytometer. The percentage of each population in total leukocytes (CD45^+^ cells) in blood, spleen and peripheral LN was determined by Flowjo software.

### 2.3 Murine model of GVHD

The animal experiments are approved by the Institutional Animal Care and Use Committee at the University of Texas MD. Anderson Cancer Center.

We used the MHC class I and II disparate murine model, C57BL/6 (H‐2D^b^) to BALB/c (H‐2D^d^), which is an established model of GVHD^26^ to test the effect of ADAMTS13 and VWF‐A2 protein on the severity of GVHD. Recipient mice were females and 8–10 weeks of age at the time of bone marrow transplantation (BMT). The single‐cell suspensions of bone marrow cells and splenocytes were prepared in PBS for injection. To generate BMT chimeras, recipient BALB/c mice received 800 cGy TBI (^137^Cs source) on Day −1. The mice then received 5 × 10^6^ bone marrow cells and 10 × 10^6^ splenocytes from C57BL/6 donor mice on Day 0. A group of recipient mice received recombinant VWF‐A2, another group received recombinant ADAMTS13 (rADAMTS13), and the control group received buffer (PBS). VWF‐A2 was administrated intraperitoneally (i.p.) at the dose of 3 mg/kg (based on our previous study with VWF‐A2 administration to a murine model of traumatic brain injury^27^), starting the same day as the bone marrow transplant (Day 0), every other day in the first week, and every 3 days in the second and third‐week post‐transplant. In our previous study with ADAMTS13 in a murine model of traumatic brain injury, we administered 200 mg/kg of ADAMTS13 to each mouse. In the current study, we compared the impact of various doses of ADAMTS13 (100, 200, 300 and 400 mg/kg) on the severity of GVHD. The benefit of ADAMTS13 reached a maximum plateau at 300 mg/kg, and we used this dose of ADAMTS13 for all experiments. ADAMTS13 was administrated via the tail vein (i.v.) at a dose of 300 g/kg 2 days before (Day −2) and 3 days after transplantation (Day +3). The control group for each experiment received PBS at the same time and through the same route as the experimental reagent. Survival and clinical signs of GVHD (hair loss, hunched back and diarrhoea) were monitored daily, and mice were weighed twice a week. For histopathological analysis of GVHD target tissues, samples were collected from the skin, liver, intestine and lung and fixed in 10% formalin. The preserved tissue samples were embedded in paraffin, sectioned and stained with haematoxylin and eosin for histological examination. A murine pathologist systematically examined and evaluated tissue slides.

In a group of bone marrow transplant experiments, irradiated BALB/c mice were sacrificed 24 h after infusion of C57BL/6 splenocytes (day +1). These mice received one injection of ADAMTS13 (Day‐2), VWF‐A2 (Day 0) or PBS. Donor T cells were quantified and immunoprofiled in the spleen and secondary lymphoid organs.

### 2.4 Immunophenotyping of GVHD

The proliferation and activation of donor T cells were measured as we described.^26^, ^28^, ^29^ The recipient mice were sacrificed 7 days post‐transplant. Spleen (SP), peripheral (PLN) and mesenteric (MLN) lymph nodes and Peyer's patches (PP) were harvested, and the single‐cell suspension was stained with CD3, CD4, CD8, CD25, Foxp3 and H‐2D^b^ antibodies. Donor‐derived T cells (CD3^+^H‐2D^b+^), CD4^+^H‐2D^b+^, CD4^+^CD25^+^Foxp3^+^H‐2D^b+^ and CD8^+^H‐2D^b+^ subsets were collected and analysed by FACS. The total number of each subset recovered from tissues was determined by Flowjo software.

### 2.5 Intracellular cytokine production in donor‐derived T cells

Intracellular cytokine production of IL‐4, IL‐17 and IFN‐γ in donor‐derived CD4^+^ cells (CD4^+^H‐2D^b+^), and IL‐2 and TNF‐α in donor‐derived CD8^+^ cells (CD8^+^H‐2D^b+^) was measured using 8‐colour 10‐parameter cytokine flow cytometry. T cells harvested from the recipient mice spleen were washed and stimulated with the Leukocyte Activation Cocktail with GolgiPlug (BD Biosciences, San Jose, CA) for 5 h, the phorbol ester, PMA (Phorbol 12‐Myristate 13‐Acetate), a calcium ionophore (Ionomycin) and the protein transport inhibitor BD GolgiPlug™ (Brefeldin A). Cells were then fixed and permeabilized with Fix & Perm A/B buffer (Life Technologies, Carlsbad, CA) and assessed for the simultaneous expression of surface markers and intracellular cytokines. FACS analyses were performed using mAbs for mouse CD4, CD8, IL‐4, IL‐17, IFN‐γ, IL‐2 and TNF‐α to identify IL‐4, IL‐17, IFN‐γ producing donor‐derived CD4^+^ and IL‐2, TNF‐α producing donor‐derived CD8^+^ T cells. The GEO mean fluorescent intensity of IL‐4, IL‐17 and IFN‐γ Abs in donor‐derived CD4^+^ cells and IL‐2 and TNF‐α in donor‐derived CD8^+^ cells was analysed using FlowJo software.

### 2.6 Histopathology of GVHD

For histopathological analysis of GVHD target tissues, 7 days after transplant, samples were collected from the skin, small intestine, liver and lung of recipient mice and fixed in 10% formalin. The tissue samples were embedded in paraffin, sectioned and stained with haematoxylin and eosin. A pathologist graded tissue slides according to an established protocol, including semiquantitative scoring of immune cell infiltration.^30^

### 2.7 Measuring VWF antigen level and activity

After obtaining baseline samples (Time 0), the mice were infused with a single bolus dose of rhADAMTS‐13 at 100 μg/mouse through the tail vein. Blood samples were collected every 30 min four times through alternate eyes in anticoagulant (0.38% sodium citrate), and plasma samples were analysed for VWF:Ag (ELISA, Abcam) and VWF:CB (Technozym® VWF:CBA Collagen I ELISA, DiaPharma).

### 2.8 Mixed Lymphocyte Reaction

Splenocytes isolated from C57BL/6 (H‐2D^b^) mice (responder cells) were incubated with 3400 cGy irradiated splenocytes isolated from BALB/c (H‐2D^d^) mice (stimulator cells) at a 1:1 ratio for 7 days, in 5% CO_2_ at 37°C in the absence or presence of various concentrations of ADAMTS13 (0.01, 0.05, 0.1, 0.5, 1 and 2 μg/mL) or VWF‐A2 and control peptide (0.001, 0.01, 0.1, 1 and 10 μg/mL). Non‐stimulated splenocytes from C57BL/6 mice were used as negative controls. To determine T‐cell proliferation, responder cells were labelled with CFSE, and cell division was monitored, and total alive CD4^+^ and CD8^+^ T‐cell numbers in culture were counted after coculture by using a BD LSRFortessa X‐20 flow cytometer and resulting data analysed by FlowJo™ software.

### 2.9 In vitro binding of Jurkat cells to HUVECs and VWF

HUVECs were cultured into a slide chamber until reaching 80% confluency. In some experiments, HUVEC cells were stimulated with 25 μM of Histamine (H7125; Sigma Aldrich) to induce endothelial cell VWF release. Jurkat cells (1 × 10^5^) were added into each slide chamber containing the HUVEC cells and incubated for 15 min at 37°C. Wells were washed with PBS and examined with an inverted microscope to count the number of adhered Jurkat cells. In some experiments, prior to adding Jurkat cells, histamine‐stimulated HUVEC cells were treated with 1 μg/mL of recombinant human ADAMTS13 (6156‐AD; R&D Systems) for 10 min at room temperature. In other experiments, 1 μg/mL of recombinant VWF‐A2 fragment, CD11a antibody (MA11A10; 1:1000; Thermo Fisher) was added to the incubated cells.

For binding studies with VWF, 96‐wells were coated with 5 μg/mL of recombinant human factor VIII‐free VWF (HCVWF‐0191; Prolytix) overnight at 4°C. The next day, wells were washed three times with 0.1% Tween20‐PBS solution. Jurkat cells either pre‐stimulated with 200 ng/mL of CCL21 (300‐35A; Pepro Tech) for 1 h at 37°C or resting were added to each well. In some wells, recombinant ADAMTS13, recombinant VWF‐A2 or anti CD11a antibody was added to the incubated Jurkat cells. After 15 min at 37°C incubation period, wells were washed with PBS and examined with an inverted microscope to count the number of adhered Jurkat cells.

### 2.10 Surface plasmon resonance (SPR) analysis

Direct binding of integrin αL chain with VWF or vWF‐A2 was measured by SPR using a Biacore 3000 optical biosensor (GE Healthcare) at 25°C. The protein sensor surface was prepared using CM5 sensor chip (GE Healthcare) by amine‐coupling chemistry (5 min EDC/NHS activation, 5 or 6 min ligand injection and 4 min deactivation with ethanolamine). During immobilization, PBST (8.06 mM Na_2_HPO_4_ and 1.94 mM KH_2_PO_4_, pH 7.4, 137 mM NaCl, 2.7 mM KCl, 0.02% Tween20) was used as a running buffer at a flow rate of 10 μL/min. Frozen stock of factor VIII‐free VWF (0.24 mg/mL in 25 mM sodium citrate, 100 mM NaCl, 100 mM Glycine, pH 6.8. **Haematologic Technologies,** catalogue # **HCVWF‐0191)** was thawed at 37°C and diluted in 10 mM sodium acetate (pH 5.0) to 24 ug/mL for immobilization. After deactivation, VWF (~3600 RU) surface density was reduced to ~3000 RU by treating with TBS (pH 8.0) containing 10 mM TCEP for 5 min to remove non‐covalently coupled VWF molecule. The stock solution of VWF‐A2 (0.6 mg/mL in TBS, 0.05% Tween‐20) was diluted in 10 mM sodium acetate (pH 5.5) to 30 μg/mL for immobilization. The surface was then treated with 0.1% SDS to remove loosely bound A2 (final density ~ 1200 RU). Integrin αL stock solution (0.16 mg/mL in 25 mM Tris, 100 mM glycine, 10% glycerol, pH 7.3. Origene, catalogue # TP321297) was diluted to 16 ug/mL in 10 mM sodium acetate (pH 5.0) for immobilization (final surface density ~ 500 RU). A reference surface was made with activation and deactivation steps but with no protein‐coupled. Binding experiments were performed at a flow rate of 30 μL/min in TBSTG (25 mM Tris, pH 7.5, 150 mM NaCl, 0.05% Tween, 2% glycerol). To regenerate the ligand surface, the bound protein was removed with a 10‐s injection of 0.1% SDS. All SPR responses were baseline and buffer corrected.

### 2.11 Statistical analysis

All statistical analyses were performed using GraphPad Prism 9 Software. We examined the differences between treatment groups, and survival data were plotted by the Kaplan–Meier method and analysed by the log‐rank test. A semiquantitative scale from 0 to 4 was used for histopathological changes by a pathologist. Pathology scores were evaluated with Fisher's exact test. Murine in vivo data was analysed using a two‐tailed Student's *t*‐test. Jurkat cell adhesion in vitro data was analysed using one‐way ANOVA test with Dunnett's multiple comparison correction. Results are presented as the mean and standard error of the mean. Statistical significance was considered for *p* ≤ 0.05.

## 3 RESULTS

### 3.1 ADAMTS13 reduced the mortality and morbidity of GVHD in mice

**FIGURE 1:**
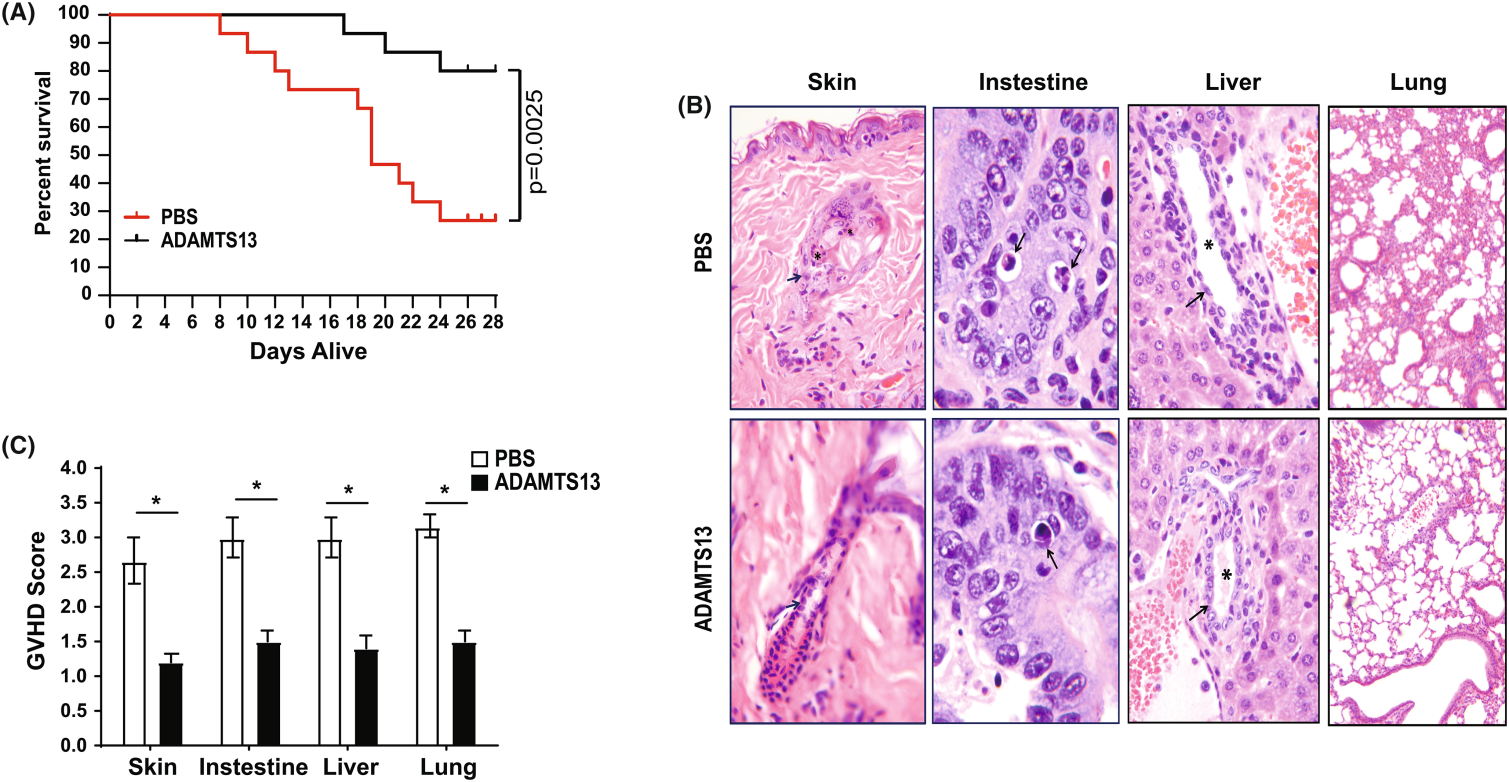
ADAMTS13 reduced GVHD in mice. ADAMTS13 treatment (at 300 μg/kg) reduced mortality and morbidity in acute GVHD in mice. (A) Survival was monitored daily for 4 weeks, with results from three independent experiments (*p* = 0.0025, *n* = 15 for each group). (B) Mice were sacrificed on Day 7 post‐transplant (five mice per group). The top and lower panels represent the skin, small intestine and liver of mice treated with PBS control and ADAMTS13, respectively. Arrows and asterisks indicate the histopathologic changes in the skin (inflammatory and apoptotic cells in hair follicles), intestine (necrosis in the crypts), liver (lymphoid infiltration in portal tracts and epithelial damage of bile duct) and lung (lesions with inflammatory infiltration). (C) Average GVHD score of skin, intestine, liver and lung. Results are shown as mean ± SD (*t*‐test; *n* = 5 in each group). *p < 0.05.

BALB/c recipient mice were irradiated and infused with bone marrow cells and splenocytes from C57BL/6 donor mice to induce GVHD (Day 0). Two days before irradiation (Day −2) and 3 days after infusion of donor cells (Day +3), a group of BALB/c recipient mice received recombinant ADAMTS13 (300 g/kg) through the tail vein. A control group of recipient mice received the same volume of PBS through the tail vein. ADAMTS13‐treated recipient mice had a significantly lower mortality rate than the vehicle PBS‐treated recipient mice (Figure 1A). At 4 weeks after BMT, 80% of ADAMTS13‐treated recipients survived, compared to 26.7% of PBS‐treated control mice (*p* = 0.0025; *n* = 15 in each group). Whereas PBS‐treated recipients had severe GVHD in the skin, intestine, liver and lung, ADAMTS13‐treated mice exhibited only mild changes in these organs (Figure 1B) and significantly lower GVHD scores (Figure 1C).

We conducted an additional experiment to measure the effect of recombinant ADAMTS13 injection on VWF antigen level (VWF:Ag) and activity (collagen‐binding activity or VWF;CB) in mice. ADAMTS13 administration did not alter VWF antigen level but reduced VWF collagen‐binding activity (Figure SS1).

### 3.2 ADAMTS13 reduced donor‐derived T‐cell number and Th17 polarization after transplant in mice

**FIGURE 2:**
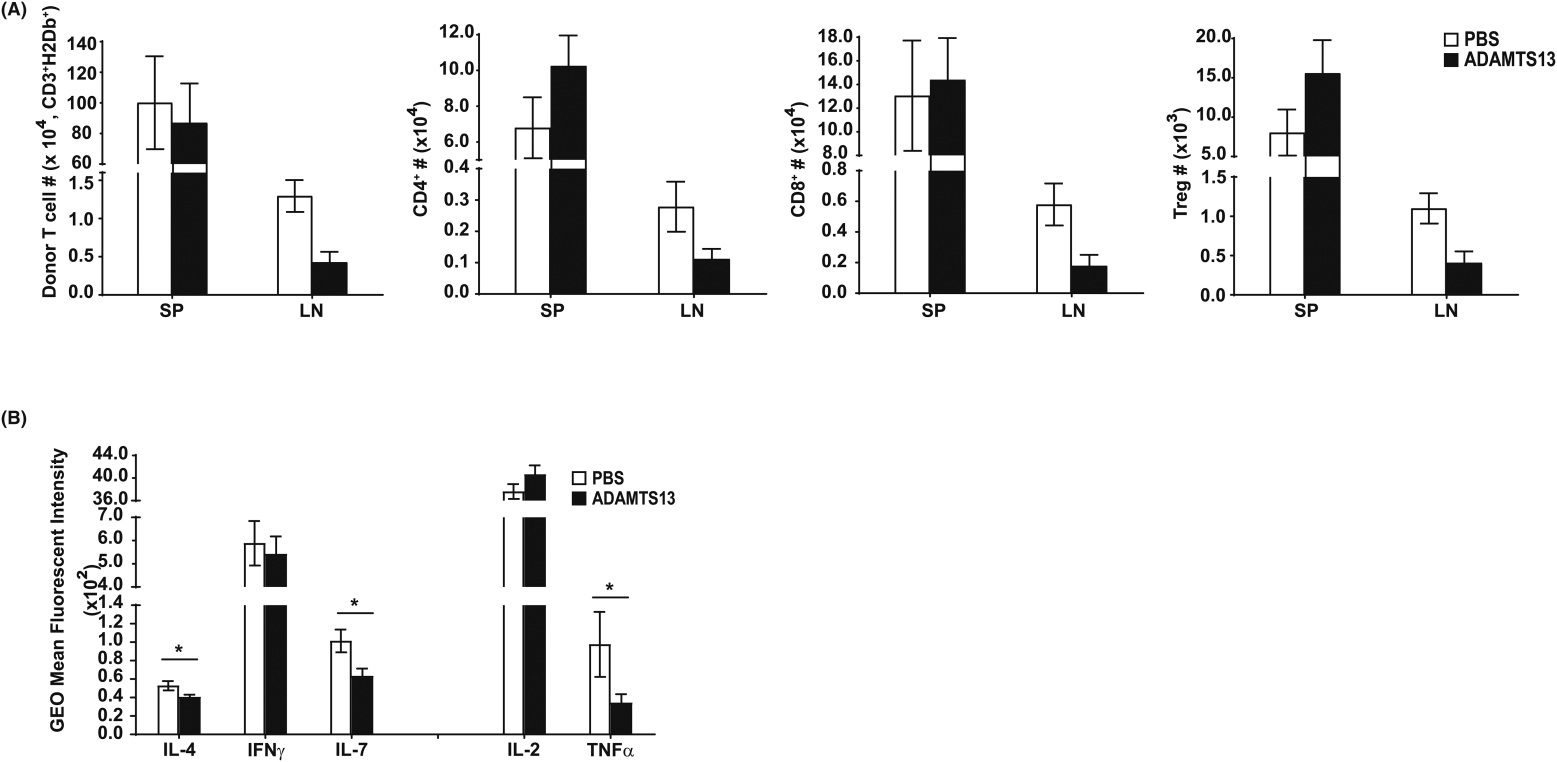
ADAMTS13 altered the number and polarization of donor‐derived T cells in mice. Mice were sacrificed on Day 7 post‐transplant (three mice per group). Cells were collected from the spleen (SP) and peripheral lymph nodes (LN) and then stained with CD3, CD4, CD8, H‐2D^b^, Foxp3 and CD25 antibodies. (A) The number of donor‐derived T‐cell subsets recovered from each tissue, CD4^+^/H‐2D^b+^, CD8^+^/H‐2D^b+^ and CD4^+^/CD25^+^/Foxp3^+^/H‐2D^b+^ (Treg) were determined by FACS. (B) Intracellular expression of IL‐2, IL‐4, IL17, IFNƔ and TNF‐α in donor‐derived T cells was compared between ADAMTS13‐ and PBS‐treated recipient mice. Results were calculated as the mean and SD of at least three independent experiments. *p*‐values were calculated using a *t*‐test comparing ADAMTS13‐treated and PBS control mice. Results are shown as bar graphs representing means ± SD. *p < 0.05.

The number and polarization of donor T cells were compared in LN and spleen of ADAMTS13‐ and PBS‐treated mice. ADAMTS13 reduced all subsets of donor‐derived T cells (CD4, CD8 and Treg) in LN of transplanted mice (Figure 2A). Measurement of the intracellular cytokines in T cells showed that ADAMTS13 reduced Th17 (IL‐17) polarization more significantly than Th2 (IL‐4) and did not affect Th1 (IFN‐Ɣ) response (Figure 2B). Administration of ADAMTS13 reduced the T‐cell response after transplanting mice (Figure 2B).

The number of T cells in the spleen reflecting the perfusion of the spleen by donor splenocytes was similar between experimental arms.

### 3.3 VWF‐A2 reduced the mortality and morbidity of GVHD in mice

**FIGURE 3:**
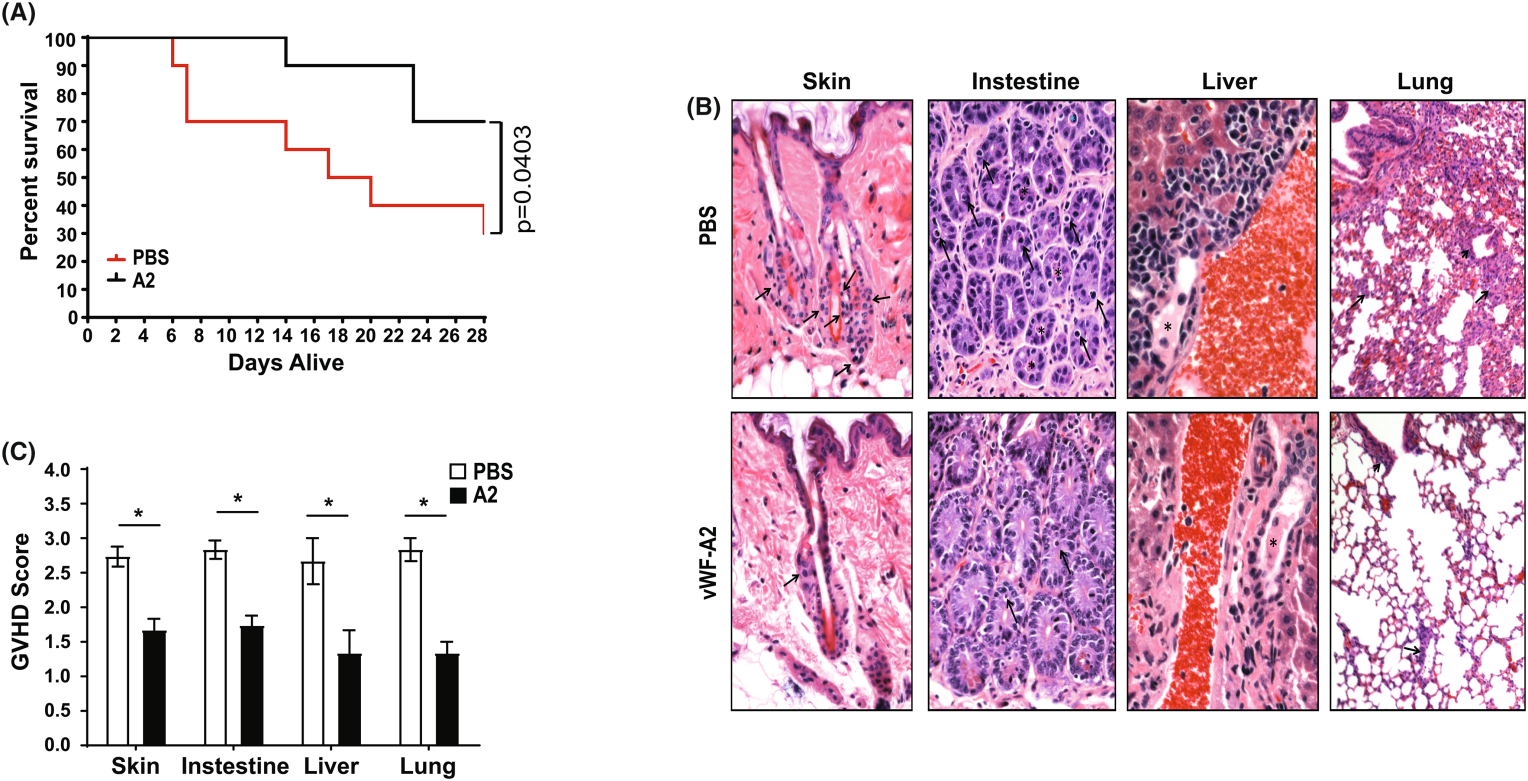
VWF‐A2 reduced GVHD in mice. VWF‐A2 treatment (3 mg/kg) significantly reduced GVHD mortality and morbidity. (A) Survival was monitored daily for 4 weeks. Distributions of time to death were estimated using the Kaplan–Meier method, and groups were compared using the log‐rank test. The results of two independent experiments (five mice/group/experiment) were summarized (*p* = 0.0403, *n* = 10 for each group). (B) Mice were sacrificed on Day 7 post‐transplant (five mice per group). The top and lower panels are representative sections from the skin, small intestine, liver and lung of mice treated with PBS control and VWF‐A2, respectively. Inflammation and tissue damage were shown in the skin (arrows represent inflammation cells in the basal cell layer of hair follicles), intestine (arrows represent single‐cell necrosis in the crypts *), liver (stars represent epithelial cells in the bile duct) and lung (arrows represent inflammation cells in blood vessel and bronchus). (C) Average GVHD scores of skin, small intestine, liver and lung in mice treated with PBS control and VWF‐A2, respectively. Results are shown as mean and SD. P values were calculated using a *t*‐test comparing PBS‐ and VWF‐A2‐treated mice. *p < 0.05.

BALB/c mice were irradiated and infused with bone marrow cells and splenocytes from C57BL/6 donors to induce GVHD (Day 0). VWF‐A2 was administrated intraperitoneally (i.p.) at 3 mg/kg, starting the same day as the bone marrow transplant (Day 0), every other day in the first week, and every 3 days in the second and third‐week post‐transplant. A control group of recipient mice received the same volume of PBS. VWF‐A2‐treated recipient mice had a significantly lower mortality rate than PBS‐treated recipients (Figure 3A). At 4 weeks after bone marrow transplant, 70% of VWF‐A2‐treated recipients survived, compared to only 30% of PBS‐treated controls (*p* = 0.0403; *n* = 10 in each group). In addition, treatment with VWF‐A2 significantly reduced GVHD‐related tissue injury in the skin, liver, small intestine and lung, as represented in Figure 3B. Average GVHD scores of skin, small intestine, liver and lung in mice treated with PBS control and VWF‐A2‐treated mice are shown in Figure 3C.

**FIGURE 4:**
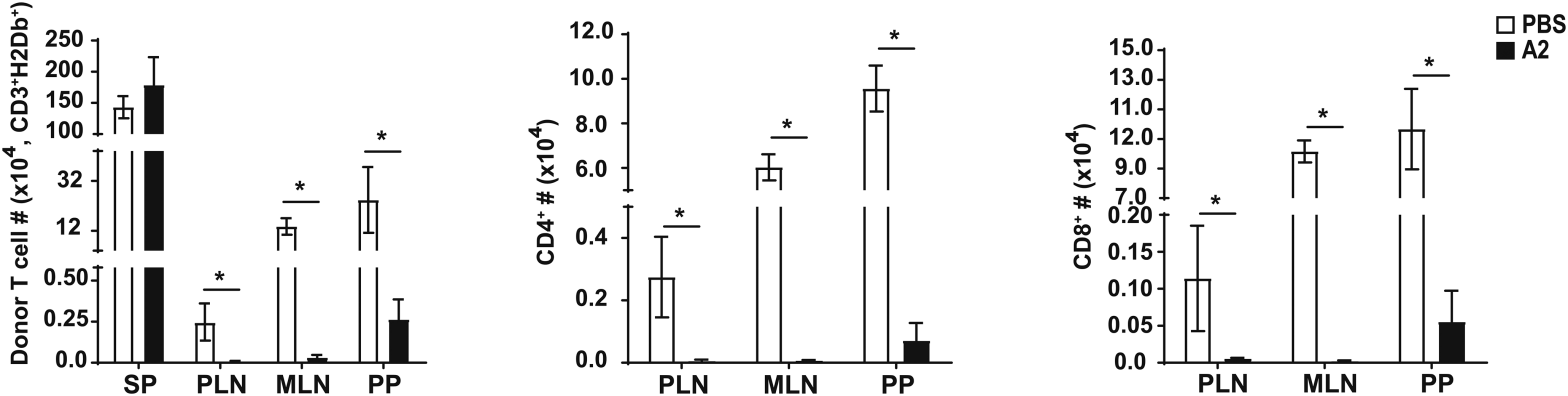
VWF‐A2 reduced donor‐derived T cells in secondary lymphoid organs. Tissues were harvested from VWF‐A2‐treated and PBS control recipient mice (*n* = 3). Cells were stained with CD3, CD4, CD8 and H‐2D^b^ antibodies. The number of donor‐derived T cells (CD3^+^H‐2D^b+^) (A), (CD4^+^H‐2D^b+^) (B) and (CD8^+^H‐2D^b+^) (C) subsets was determined by FACS. Results are shown as bar graphs representing means and SD. *p*‐values were calculated using a *t*‐test comparing A2‐treated and PBS control mice. *p < 0.05.

We quantified the number of donor‐derived T cells in the spleen and lymphoid nodes (peripheral and mesenteric LN and Peyer's patches) of recipient mice using flow cytometry, as described in the material and methods. VWF‐A2 significantly reduced the number of donor‐derived T cells (CD4 and CD8 subsets) in the secondary lymphoid tissue of recipient mice (Figure 4).

**FIGURE 5:**
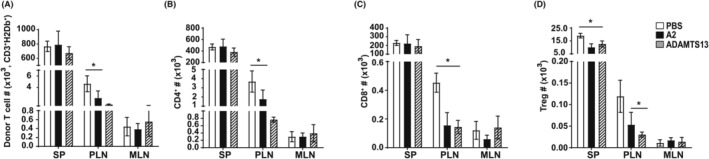
ADAMTS13 and VWF‐A2 reduced donor‐derived T cells in secondary lymphoid organs 24 h after bone marrow transplant. Mice were sacrificed 24 h post‐transplant (*n* = 4). Cells were collected from the spleen (SP), peripheral lymph nodes (PLN) and mesenteric lymph nodes (MLNs) and then stained with H‐2D^b^, CD3, CD4, CD8, CD25 and Foxp3 antibodies. The number of donor‐derived T cells and their subsets recovered from each tissue are shown. (A) The total number of donor‐derived T cells (CD3^+^H‐2D^b+^), (B) CD4+ (CD4^+^/ CD3^+^/H‐2D^b+^), (C) CD8+ (CD8^+^/CD3^+^/H‐2D^b+^) and (D) Tregs (CD4^+^/CD3^+^/CD25^+^/Foxp3^+^/H‐2D^b+^) was determined by FACS. Results are shown as bar graphs representing mean ± SD. *p‐*values were calculated using a *t*‐test comparing ADAMTS13‐treated or VWF‐A2‐treated and PBS‐treated (control) mice. *p < 0.05.

To eliminate (or reduce) the effect of variables besides homing of donor lymphocytes on the number of T cells in the spleen and LN of recipient mice, we quantified T cells 24 h after bone marrow transplantation. These mice received one injection of ADAMTS13 (Day‐2), VWF‐A2 (Day 0) or PBS. We found that ADAMTS13 and VWF‐A2 significantly reduced the number of donor T cells (both CD4+ and CD8+ cells) in the secondary lymphoid organs of recipient mice compared to the control (Figure 5). Treg numbers were significantly reduced in both spleen and LN (PLN) with ADAMTS13 and VWF‐A2 treatment compared to PBS control treatment.

### 3.4 ADAMTS13 and VWF‐A2 did not impact T‐cell proliferation in vitro

**FIGURE 6:**
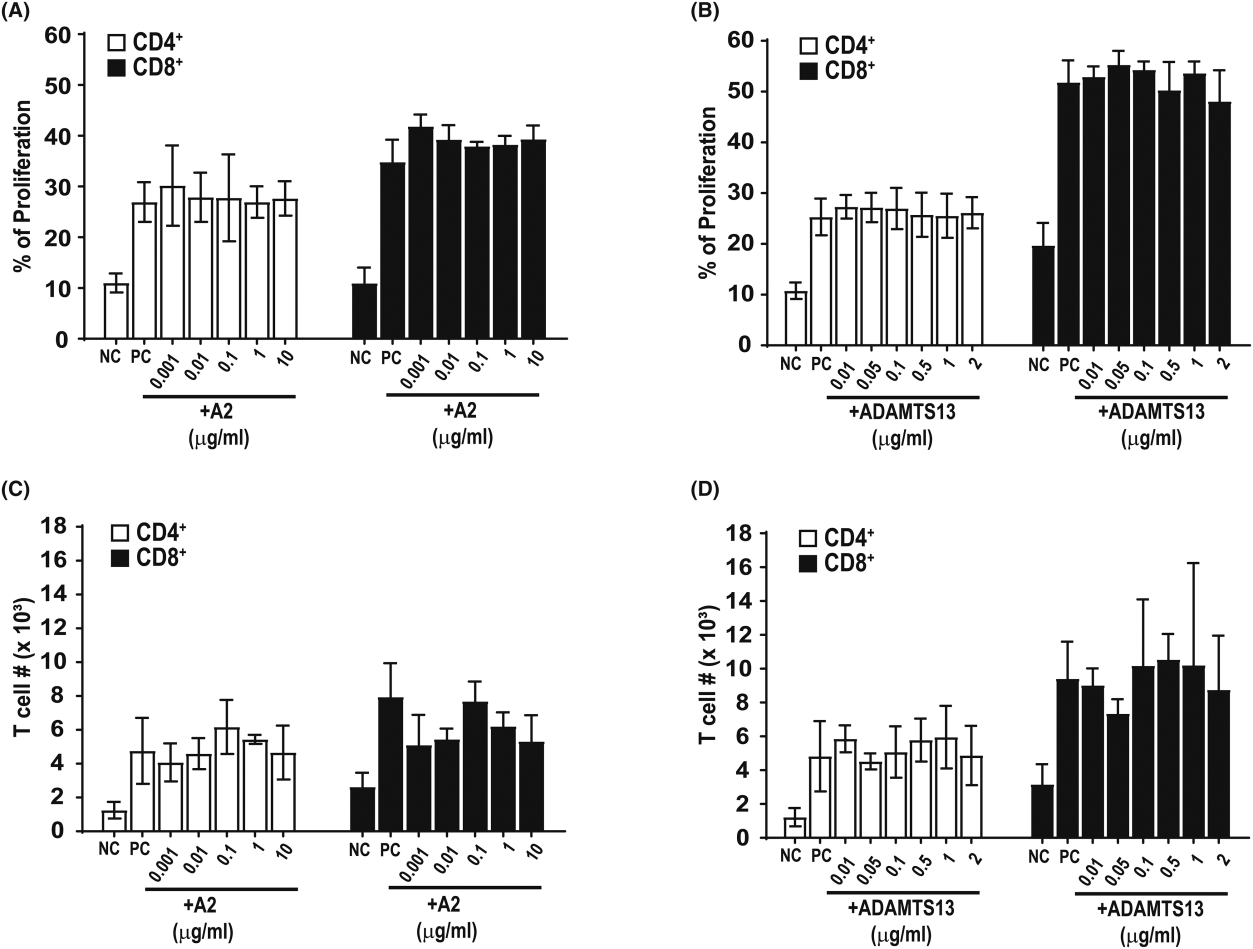
ADAMTS13 and VWF‐A2 did not impact T‐cell proliferation in vitro. CFSE‐labelled C57BL/6 responder cells were cocultured at a ratio of 1:1 with irradiated Balb/C stimulator cells for 7 days at 37°C. Primary T‐cell proliferation percentage was assessed by CFSE dye dilution following sequential gating on antibody‐stained (A) CD4 ^+^ or (B) CD8^+^ T cells. The number of alive (C) CD4 ^+^ and (D) CD8 ^+^ T cells was averaged from three independent experiments in MLR. Results are shown as bar graphs representing mean ± SD. *p‐*values were calculated using a *t*‐test. *p < 0.05. NC: negative control, C57BL/B6 responder splenocytes only (unstimulated T cells). PC: positive control, C57BL/B6 responder splenocytes cocultured with irradiated Balb/C stimulator splenocytes at 1:1 ratio.

We examined the impact of ADAMTS13 and VWF‐A2 on T‐cell proliferation in vitro in mixed lymphocyte reaction (MLR) using CFSE‐labelled splenocytes from C57BL/6 mice and MHC‐mismatched irradiated splenocytes from BALB/C mice. Various concentrations of ADAMTS13 and A2 did not increase or decrease T‐cell proliferation percentages (Figure 6 A and B) or total alive T‐cell numbers (Figure 6 C and D) in MLR (neither CD4+ nor CD8+ cells). These results are consistent with the lack of a direct impact of ADAMTS 13 and A2 on the proliferation or survival of T cells.

### 3.5 Impact of ADAMTS13 and VWF‐A2 on number and distribution of white blood cells

We performed complete blood counts and extensive analysis of white blood cells and their immune profile in blood, spleen and LN of BALB/C mice before and on Days 4 and 7 after administration of VWF‐A2 and ADAMTS13. VWF‐A2 and ADAMTS13 did not change white blood cell counts, haemoglobin or platelet counts at any time points (Figure S2). An extensive cytometric immune profiling did not show a significant difference in various subgroups of T lymphocytes in blood, spleen and LN (Figure S3). ADAMTS13‐treated mice showed a significantly lower percentage of neutrophils in blood on Day 4 after treatment but no difference on Day 7. ADAMTS13‐treated mice showed a significantly lower percentage of dendritic cells in blood on Day 7 after treatment. VWF‐A2 treated mice showed a significantly higher percentage of B cells from LN on Day 7 after treatment.

### 3.6 Jurkat cells bind to endothelial cells and VWF

**FIGURE 7:**
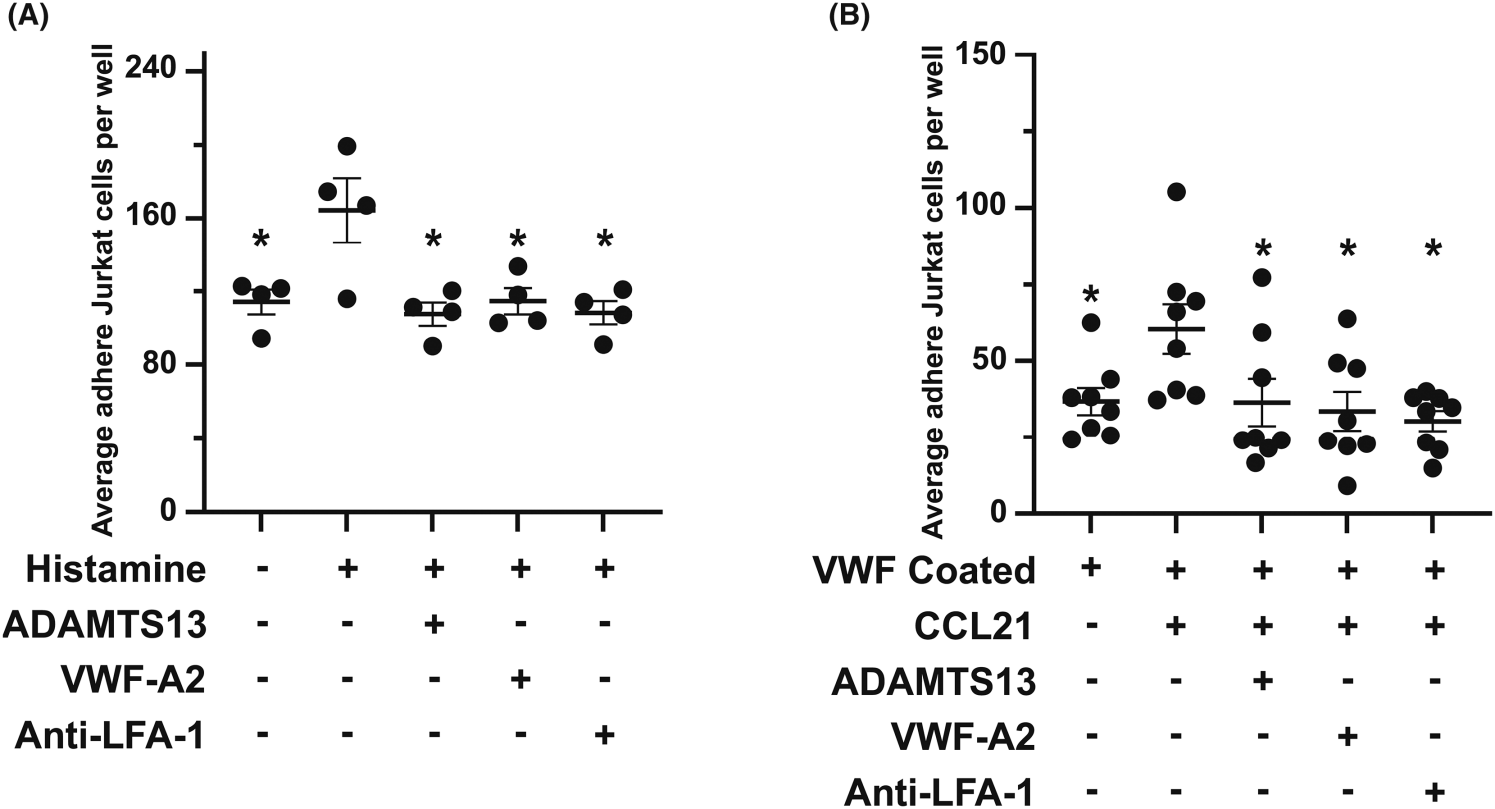
Role of VWF in the binding of T cells. (A) One hundred thousand Jurkat cells (immortalize malignant T lymphocytes) were incubated with HUVECs cells plated on 6‐well plates. After 15 min incubation at 37°C, the number of adhered Jurkat cells in each well was counted using pictures taken with an inverted microscope and compared between histamine‐stimulated and non‐stimulated HUVECs. The effect of recombinant ADAMTS13 (1 mg/mL), recombinant VWF‐A2 (1 mg/mL) and anti‐α_L_ antibodies (1:1000 dilution) on the number of adhered Jurkat cells to histamine‐stimulated HUVECs were compared (*n* = 4, each experiment in triplicates). *p‐*values were calculated using a one‐way ANOVA test with Dunnett's multiple comparison correction compared to the number of adhered Jurkat cells to histamine‐stimulated HUVECs. * *p* < 0.05. (B) One hundred thousand Jurkat cells were incubated in VWF‐coated 96‐wells plates for 15 min at 37°C. Jurkat cells, either pre‐stimulated with 200 ng/mL of CCL21 or resting, were added to each well. The effect of recombinant ADAMTS13, recombinant VWF‐A2 and anti‐αL antibodies on the number of adhered CCL21‐stimulated Jurkat cells to VWF was compared (*n* = 8, each experiment in triplicates). *p*‐values were calculated using a one‐way ANOVA test with Dunnett's multiple comparison correction compared to the number of adhered CCL21‐stimulated Jurkat cells VWF. *p < 0.05.

We used an in vitro binding assay to examine the impact of ADAMTS13 and VWF on the binding of Jurkat cells (immortalized human T cells) to human umbilical vein endothelial cells (HUVECs) (Figure 7A). Stimulation of HUVECs with histamine results in the expression of adhesion molecules and release of VWF from HUVECs and increased binding of Jurkat cells to HUVECs (from 114 ± 6 Jurkat cells per well in non‐stimulated HUVECs to 164 ± 17 per well in histamine‐stimulated HUVECs, *p* = 0.009). ADAMTS13 reduced the binding of Jurkat cells to stimulated HUVECs (107 ± 6 per well, *p* = 0.003). Recombinant VWF‐A2 and anti‐αL antibodies also reduced the binding of Jurkat cells to stimulated HUVECs significantly (114 ± 7 per well, *p* = 0.009; 108 ± 6, *p* = 0.004, respectively).

These results point towards the involvement of VWF in binding Jurkat cells to HUVECs. However, to directly examine the binding of T cells to VWF, we incubated Jurkat cells on VWF‐coated surfaces (Figure 7B). The binding of T cells to endothelial cells is a multistep process and requires adhesion molecules and chemokines. Firm adhesion of T cells is mediated by integrins that are activated via an inside‐out signalling initiated by chemokine receptors on T cells. Among chemokine receptors on T cells, CCR7 plays an important role in integrin activation and firm adhesion. In our in vitro assay, incubation of Jurkat cells with CCL21 (chemokine activating CCR7) increased binding to VWF from 36 ± 4 cells per well to 60 ± 8 cells (*p* = 0.038). Coincubation with recombinant ADAMTS13, recombinant VWF‐A2, or anti‐αL antibodies reduced binding of CCL21‐activated Jurkat cells to VWF (36 ± 7 cells per well, *p* = 0.035; 33 ± 6 cells per well, *p* = 0.016; and 30 ± 3 cells per well, *p* = 0.006, respectively).

### 3.7 αL binds to VWF and VWF‐A2 binds to αL

**FIGURE 8:**
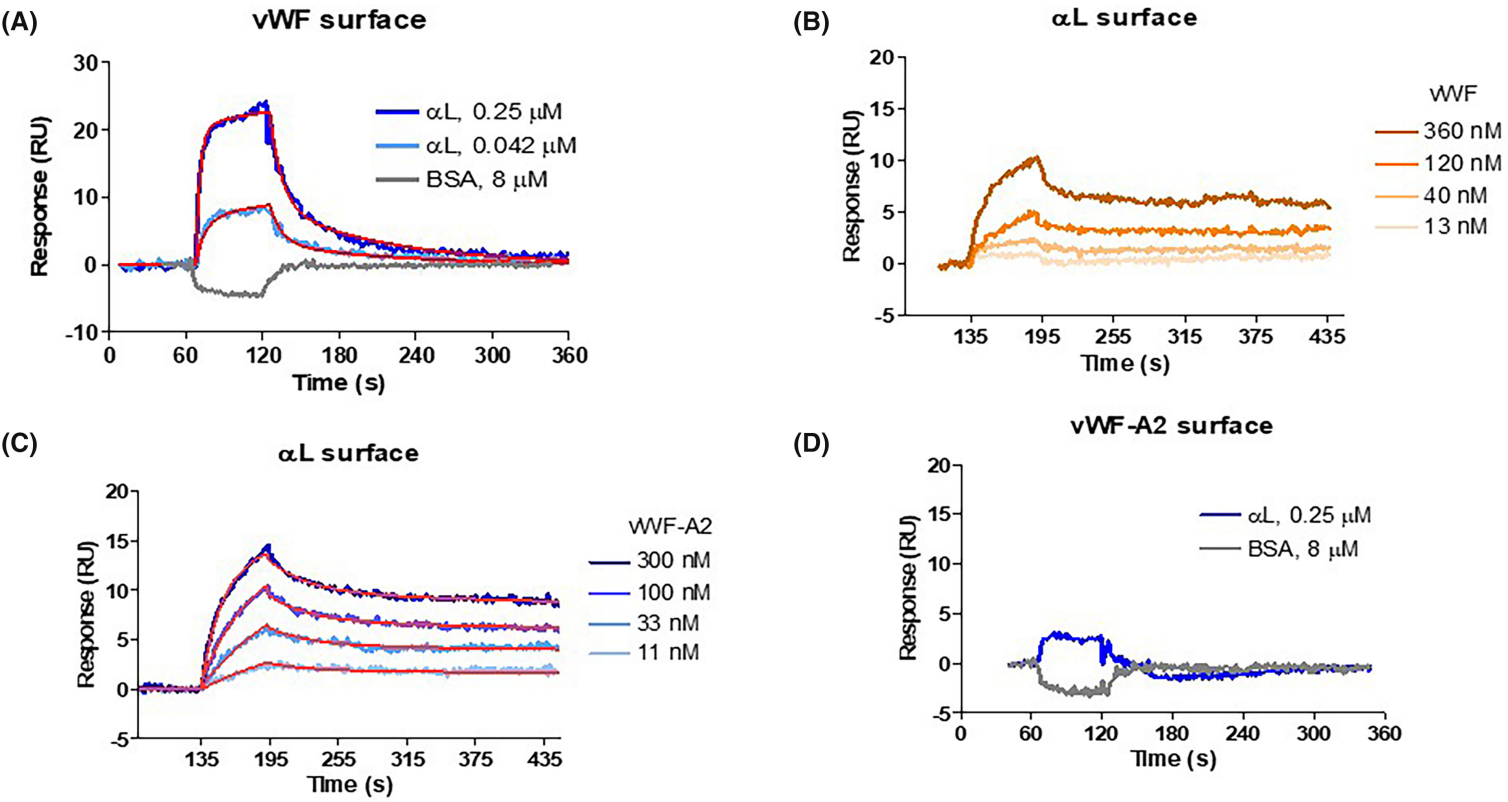
Surface plasmon resonance studies on the interaction between al and VWF and VWF‐A2. The presence and dynamic of binding of integrin αL chain to VWF or VWF‐A2 was examined using a Biacore. (A) At a flow rate of 30 μL/min, binding of α_L_ to immobilized factor VIII‐free VWF (final density of ~3000 RU) showed an apparent dissociation constant of *K*_D_ = 0.12 μM calculated from the rate constants (*k*_a1_ = 8.2 × 10^5^ M^−1^ s^−1^ and *k*_d1_ = 9.9 × 10^−2^ s^−1^; *K*_D=_ *k*_d1/_ *k*_a1_). (B) The binding constant for soluble VWF to immobilized αL (~500 RU) surface could not be uniquely determined through kinetics analysis. (C) Soluble VWF‐A2 binds to immobilized αL (~500 RU) with an apparent *K*_D_ value of 94 nM (*k*_a1_ = 1.4 × 10^5^ M^−1^ s^−1^ and *k*_d1_ = 1.3 × 10^−2^ s^−1^). (D) Minimal binding of αL to VWF‐A2 surface (~1200 RU) could be detected compared to BSA, but the affinity could not be measured.

We examined the interaction between VWF and αL and between VWF‐A2 and αL using SPR (Figure 8). We found that αL binds to immobilized VWF with an apparent dissociation constant of *K*_D_ = 0.12 μM calculated from the rate constants (*k*_a1_ = 8.2 × 10^5^ M^−1^ s^−1^ and *k*_d1_ = 9.9 × 10^−2^ s^−1^; *K*_D=_ *k*_d1/_ *k*_a1_) (Figure 8A). Sensorgrams for αL binding to immobilized VWF (~3000 RU) were fitted to a two‐state binding model (the fitted curves are shown in red) for this complex interaction due to heterogeneous VWF molecules. The binding constant for soluble VWF to αL surface could not be uniquely determined through kinetics analysis. However, the interaction was concentration‐dependent (the molar concentration of VWF was determined using monomeric MW of 260 kDa) (Figure 8B). Soluble VWF‐A2 binds to immobilized αL (~500 RU) (Figure 8C) with an apparent *K*_D_ value of 94 nM (*k*_a1_ = 1.4 × 10^5^ M^−1^ s^−1^ and *k*_d1_ = 1.3 × 10^−2^ s^−1^). Minimal binding of αL to VWF‐A2 surface (~1200 RU) could be detected at 250 nM (compared to non‐binder BSA at 8 μM), but the affinity could not be measured (Figure 8D).

## 4 DISCUSSION

The role of endothelial cells and adhesion molecules in the migration and homing of leukocytes have been extensively studied in infection and inflammation but less so in GVHD.^31^ Endothelial cells in lymphoid and non‐lymphoid organs are the first cells exposed to alloreactive T cells infused during allo‐HSCT.^32^, ^33^ Adhesion molecules on endothelial cells (high endothelial venules in LN) interact with circulating blood cells (neutrophils, monocytes, platelets and lymphocytes). These interactions mediate overlapping steps of tethering, rolling, firm adhesion and extravasation that are remarkably similar in T cells and other blood cells (neutrophils and platelets).^34^ After intravenous infusion of graft containing haematopoietic stem cells, T cells reach endothelial cells in various lymphoid and non‐lymphoid organs.^3^ The initial activation‐independent interaction between T cells and high endothelial venules (HEVs) is between L‐selectin on T cells and peripheral lymph node addressins (PNAds) mediating tethering and rolling T cells. Activation of T cells by chemokines (e.g., CCL21 and CCL19) via G‐protein‐coupled receptors (e.g., CCR7) results in activation‐dependent adhesion of T cells mediated by integrins αLβ2 and α4β7 on T cells, and ICAM‐1 and mucosal addressin cell adhesion molecule 1 (MAdCAM‐1) on HEVs, respectively.^4^, ^6^ The organ‐specific combination of chemokines determines the makeup of cellular infiltration. However, there is overlap and redundancy between various chemokines and their receptors in the migration and homing of leukocytes to GVHD‐targeted and secondary lymphoid organs.^4^, ^35^ The same redundancy and specificity apply to the role of integrins in T‐cell homing. The binding of α4β7 to MAdCAM‐1 is important for homing T cells to the intestines and Peyer's patches.^36^, ^37^ The interaction between αLβ2 and ICAM‐1 is required for homing T cells to LN and lungs, but other ligands for αLβ2 may be involved in GVHD of the liver and intestines.^4^

Our current study showed that the administration of exogenous ADAMTS13 in the initial days of bone marrow transplant (Day −2 and Day +3) was adequate to have a long‐lasting impact on reducing the severity of GVHD and GVHD‐related mortality. ADAMTS13 reduced the number of T cells in secondary lymphoid organs (LN, spleen and Peyer's patches). We have previously shown exogenous ADAMTS13 did not significantly change plasma levels of VWF antigen but increased levels of cleaved VWF, reduced the adhesive activity of VWF measured by the collagen‐binding assay, reduced platelet activation measured by CD62p expression and improved outcomes of mice with traumatic brain injury^38^ and endotoxemia.^39^ We have also shown that ADAMTS13 has a reductase activity that prevents inter‐multimer disulfide bond formation without changing the overall multimer distribution.^40^, ^41^ We hypothesized that VWF is a ligand for T cells and that ADAMTS13 proteolytically reduces anchored VWF on HEVs and endothelial cells to prevent T‐cell adhesion, migration and homing. VWF is a known ligand for integrins on leukocytes, and deficiency or blocking of VWF reduces leukocyte recruitment to the site of inflammation.^7^ ADAMTS13 deficiency increases inflammation and tissue injury caused by ischemia, trauma or infection.^16^, ^18^, ^19^, ^23^, ^42^ The ratio of VWF to ADAMTS13 predicts the severity of inflammation and organ failure.^14^, ^15^ The anti‐inflammatory effect of ADAMTS13 is attributed to the reduction in the recruitment of neutrophils, monocytes and T cells.^18^, ^21^ We examined whether the decrease in the number of T cells in lymphoid organs in transplanted mice after administration of ADAMTS13 was due to a reduction in T‐cell adhesion. We used an in vitro adhesion assay by incubating Jurkat cells (immortalized malignant T cells) on HUVEC cells. Jurkat cells adhered to histamine‐stimulated HUVEC cells, and ADAMTS13 reduced this binding. We and others have shown that ADAMTS13 cleaves VWF on stimulated endothelial cells under static conditions.^43^, ^44^ The adhesion of Jurkat cells to VWF‐coated surfaces confirmed that VWF could support the adhesion of Jurkat cells. The binding of Jurkat cells to histamine‐stimulated HUVEC and immobilized VWF was abolished with an antibody to αLβ2 integrin. To further examine the binding of VWF to αLβ2, we measured the binding kinetics of recombinant αL to immobilized VWF using plasmon surface resonance (SPR) and detected a *K*_D_ = 0.12 μM.

We showed that aL binds to VWF (*K*_D_ = 0.12 μM), and the VWF‐A2 domain can bind to aL (a *K*_D_ = 94 nM). In vitro, VWF‐A2 reduced the adhesion of Jurkat cells to stimulated HUVEC cells and immobilized VWF. We hypothesized that recombinant VWF‐A2 can compete with VWF for binding to aL, reducing the binding of lymphocyte αLβ2 to VWF on endothelial cells. We examined the effect of VWF‐A2 on GVHD by intraperitoneal injection of VWF‐A2 in the first 3 weeks after bone marrow transplant in mice. Like ADAMTS13, VWF‐A2 reduced the GVHD severity, mortality and the number of T cells in lymphoid organs. In addition to our novel finding on the impact of VWF‐A2 in reducing the interaction between αLβ2 on lymphocyte and VWF on endothelial cells, VWF‐A2 has been previously shown to reduce the binding of VWF to other ligands, that is, fibrin(ogen),^45^ glycoprotein Ibα^27^ and vimentin.^46^ The recombinant peptide encompassing the VWF‐A2 domain sequence has been shown to reduce mortality in a murine model of sepsis,^45^ attenuate microvascular thrombosis in systemic inflammation^47^ and morbidity after traumatic brain injury in mouse models.^27^ We have previously shown that the VWF‐A2 binds to VWF‐A1.^48^ Therefore, it might bind to the I domain on αL that is homologous to the VWF‐A domain.

The role of αLβ2 in the migration and recirculation of lymphocytes has been established.^49^, ^50^ Our group and others have shown that blocking integrin αLβ2 (LFA‐1) reduces the severity of GVHD in vivo^28^, ^51^ and lymphocyte activation adhesion and activity in vitro.^28^, ^52^ The active conformation of the I domain of αL provides the binding site for several ligands, including ICAM‐1, ICAM‐2 and ICAM‐3. Blocking αLβ2 by antibody^53^, ^54^ or lovastatin^28^ reduces the severity of GVHD in mice and homing of T cells to lymphoid organs. In the current study, we identified VWF as a ligand for αLβ2 and propose that blocking the interaction between VWF and αLβ2 (VWF‐A2) can be a novel therapeutic tool against GVHD.

Our findings open the possibility of using ADAMTS13 or VWF‐A2 (non‐immunosuppressive reagents) to prevent GVHD. Most interventions in stem cell transplant aim to prevent GVHD, including prophylactic immunosuppression.^1^ The conditioning regimens include chemotherapy and, in many instances, radiotherapy. Both chemotherapy and radiation induce endothelial injury and VWF release. The intensity of the conditioning regimen is known to impact the severity of GVHD.^55^ We hypothesized that VWF on endothelial cells stimulated by pretransplant radiation in mice and conditioning chemotherapy in humans enhances the adhesion and migration of infused donor T cells. T lymphocytes homed to secondary lymphoid organs mount an alloimmune response to recipient antigens. Blocking binding to VWF (A2) or cleaving VWF (ADAMTS13) at the time of endothelial injury was the rationale for our experimental approach of giving ADAMTS13 before radiation and A2 at the time of transplant to target homing of T cells to reduce the severity of tissue injury in GVHD. After GVHD has developed, treatment options and the success rate are limited. Administration of ADAMTS13 or VWF‐A2 before or after conditioning might be a nontoxic and non‐immunosuppressive method to reduce subsequent GVHD severity. This speculation needs to be explored in additional studies.

The role of VWF in the GVHD is another example of the close interaction between adaptive immunity and haemostatic factors in the pathogenesis of various disorders. We speculate that ADMTS13 or VWF‐2 might have a similar therapeutic impact on other T‐cell‐mediated immune disorders.

## AUTHOR CONTRIBUTIONS

**Dan Li:** Data curation (lead); formal analysis (lead); investigation (lead). **Min Soon Cho:** Data curation (supporting); formal analysis (supporting); visualization (equal). **Ricardo Gonzalez‐Delgado:** Data curation (supporting); formal analysis (supporting); visualization (supporting). **Xiaowen Liang:** Data curation (equal); formal analysis (equal); visualization (equal). **Jing‐Fei Dong:** Conceptualization (equal). **Miguel A. Cruz:** Conceptualization (equal); resources (equal). **Qing Ma:** Conceptualization (equal); data curation (equal); formal analysis (equal); investigation (equal); methodology (equal); supervision (equal); visualization (equal). **Vahid Afshar‐Kharghan:** Conceptualization (lead); funding acquisition (lead); investigation (lead); methodology (lead); writing – original draft (lead); writing – review and editing (lead).

## CONFLICT OF INTEREST STATEMENT

Miguel A. Cruz is the Chief Scientific Officer of A2 Therpeutics Inc.; the other author(s) declare no competing financial interests.

## Supporting information


Figure S1:

## Data Availability

The data that support the findings of this study are available from the corresponding author upon reasonable request.
