# Supplementary material for: The effect of ADAMTS13 on graft‐versus‐host disease
Source: J Cell Mol Med. 2024 Jul 4;28(13):e18457. doi: 10.1111/jcmm.18457 (PMC11222974; doi:10.1111/jcmm.18457)
Supplement: Supplementary file 1 — Figure S1: [file JCMM-28-e18457-s001.docx]

**Supplementary File**


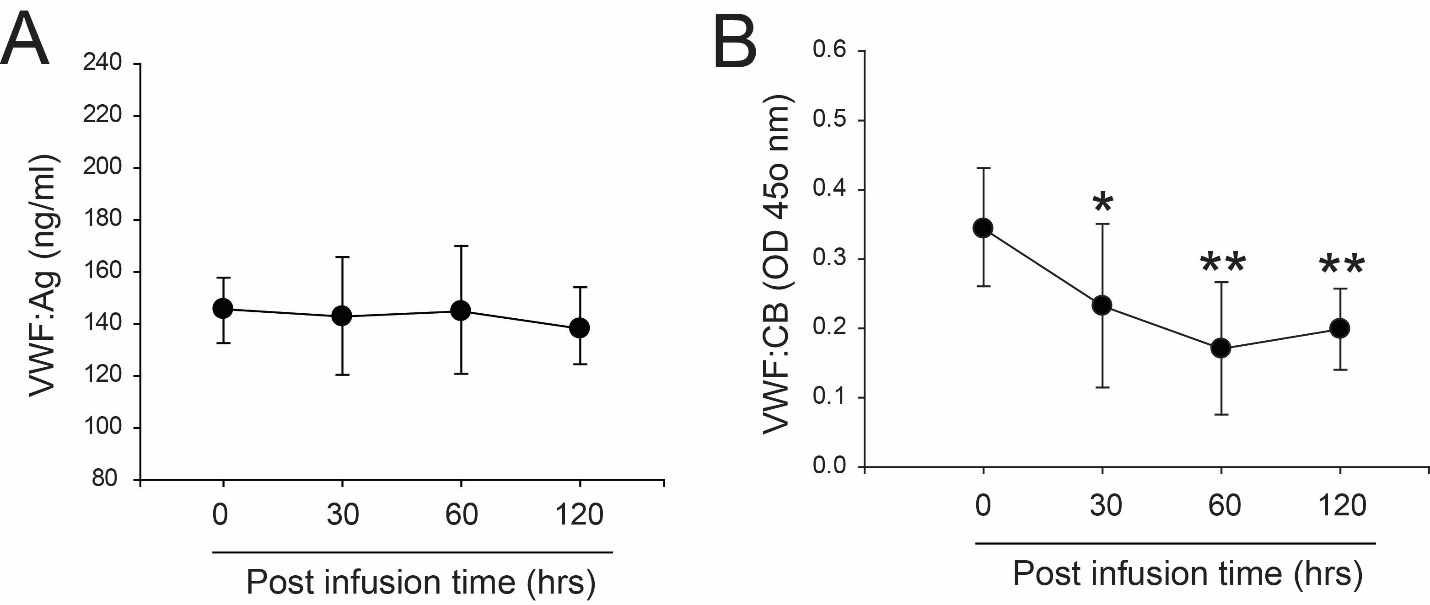


**Supplementary Figure 1**


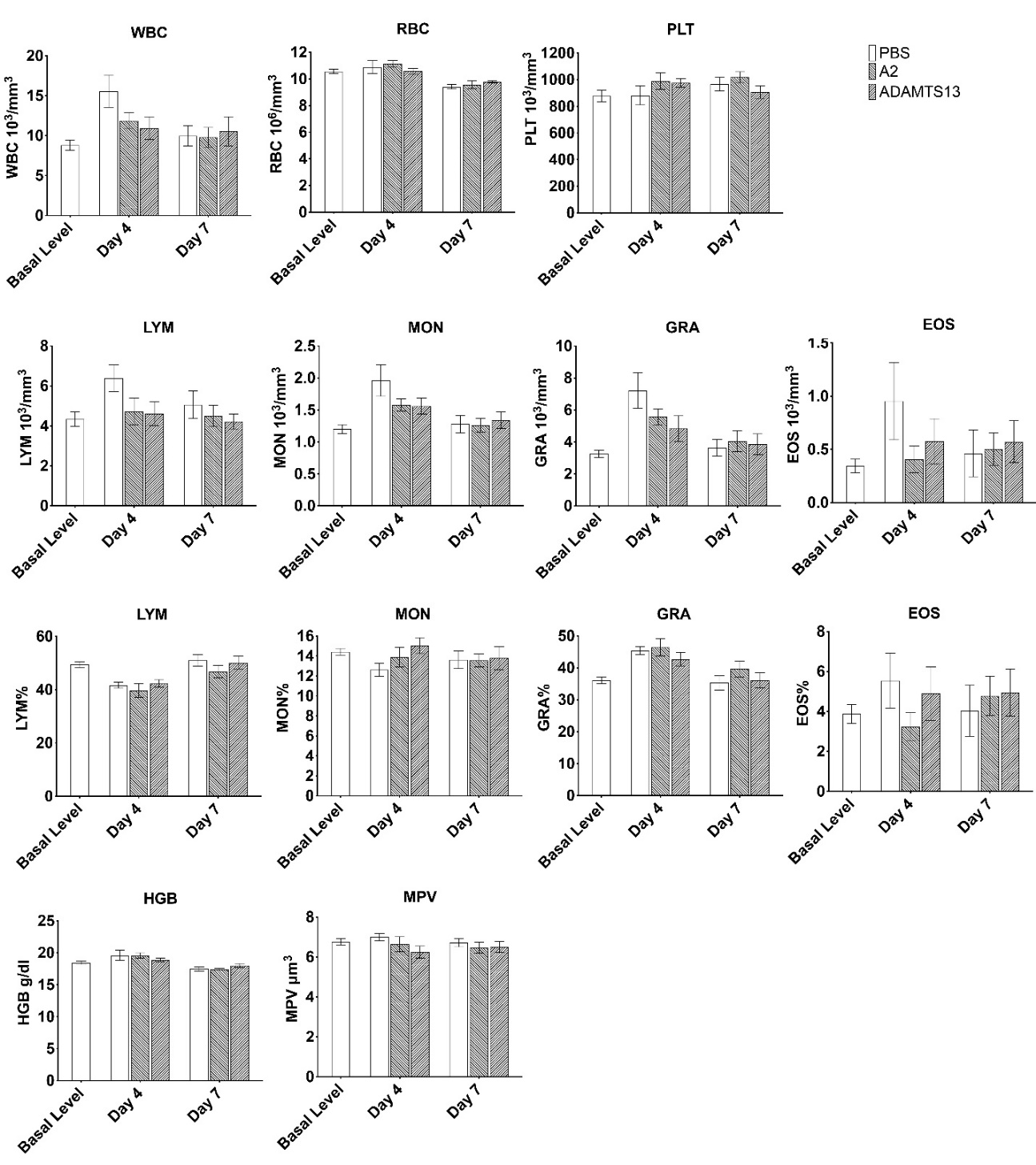


**Supplementary Figure 2**


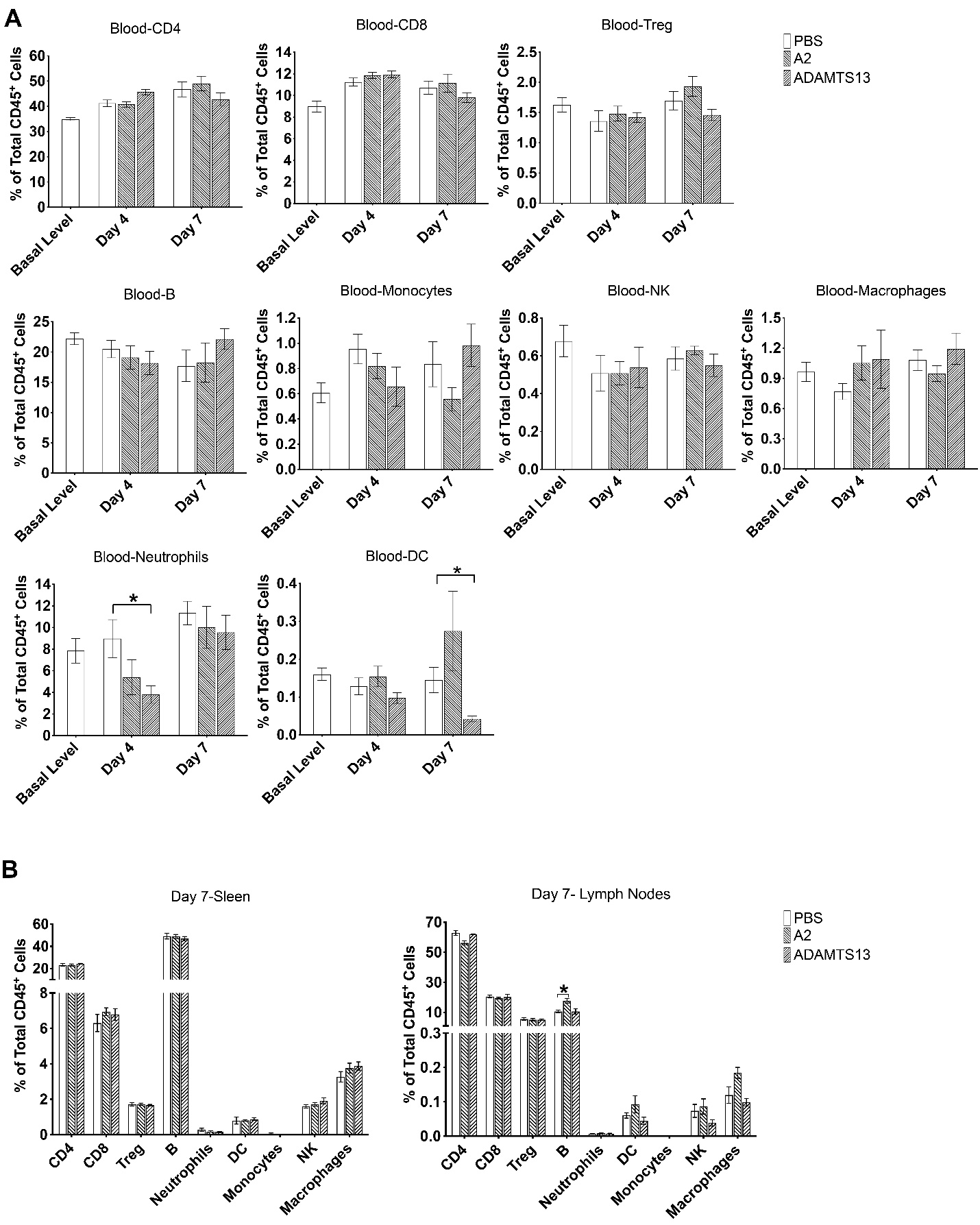


**Supplementary Figure 3**

**Supplementary Figure Legends**

**Supplementary Figure 1: Effect of recombinant human ADAMTS-13 on VWF antigen (VWF:Ag) and VWF binding to collagen (VWF:CB) in C57BL/6J mice.** After baseline samples (time 0), the mice were infused with a single bolus dose of recombinant ADAMTS-13 at 100 µg/mouse through the tail vein. Blood samples were collected through alternate eyes (0.38% sodium citrate as anticoagulant) and plasma samples were analyzed for VWF:Ag (ELISA, Abcam) and VWF:CB (Technozym® VWF:CBA Collagen I ELISA, DiaPharma). The data were analyzed with one-way ANOVA (n=6 mice, *p<0.05 vs. baseline, **p<0.05 vs. 30 min after ADAMTS-13 infusion)

**Supplementary Figure 2.** **ADAMTS13 and VWF-A2 did not impact the complete blood count in mice.** The complete blood count was performed on healthy BalbC mice before (basal level) and after ADAMTS13 and VWF-A2 treatment (day 4 and day 7). Results are shown as bar graphs representing means and SD. P values were calculated using a t-test comparing ADAMTS13 or A2-treated and PBS control mice (n=5).

**Supplementary Figure 3.** **ADAMTS13 and VWF-A2 did not impact the immune profiling in mice.** Antibodies staining and flow cytometry analysis were performed to calculate the percentage of each immune cell population (including T cells, B cells, monocytes, NK cells, macrophages, neutrophils, and DC) on day 4 and day 7: **(A)** blood from healthy BalbC mice before (basal level) and after ADAMTS13 and VWF-A2 treatment (day 4 and day 7); **(B)** Mice were sacrificed on day 7 after treatment. Cells were collected from the spleen (SP) and peripheral lymph nodes (LN). Results are shown as bar graphs representing means and SD. P values were calculated using a t-test comparing ADAMTS13 or A2-treated and PBS control mice. * p<0.05, and n=5.
